# The British Hospital at Sunny Bank, Cannes

**Published:** 1904-10-22

**Authors:** 


### THE BRITISH HOSPITAL AT SUNNY BANK, CANNES.

The presence of an English hospital in a foreign town does away with one of the greatest drawbacks felt by invalids who take up their residence in some favoured climate; and even those who are not ill derive much peace of mind from the knowledge that a well-managed institution is within reach should accident or infectious disease overtake them. We have before us the report of the hospital at Cannes for the year 1903, from which we learn that during that year 42 patients were in residence for an aggregate of 890 days; and these numbers show a considerable increase over those of the previous year, indicating not a more sickly season but a greater disposition on the part of English residents to make use of the hospital. The hospital does not confine its usefulness to Cannes, but receives critical cases from all parts of the Riviera; and it is deserving of notice that the institution admits both paying patients and free cases, in fact the latter reached the respectable figure of 225 days of free residence. The income from the endowment of free beds amounted to 1,505 francs, and the board state that they expended nearly double the endowment on these free beds. Analysing the 42 cases under treatment during 1903, we find that they included 16 medical, 12 surgical, and 14 infectious or contagious cases. As regards the surgical cases they can now be most satisfactorily treated as Mr. Homan has presented the hospital with an operating-theatre and another ward; but as regards the infectious cases, the accommodation in the hospital does not permit of complete isolation of these cases from each other should different infectious diseases be admitted about the same time. As a further point it is stated that patients suffering from infectious diseases are conveyed to Sunny Bank in hired carriages, and therefore an ambulance is an urgent necessity.

Among the list of subscribers we notice several well-known names, and we cannot doubt that many others will subscribe handsomely when they see the good which the hospital is doing with its limited income, and the greater boon it would confer on English residents in the Riviera if it had more money at its disposal. Subscriptions may be sent to the Secretary, Mr. Cecil H. Wybergh, at the hospital.

